# Targeted Delivery of Bioactive Molecules for Vascular Intervention and Tissue Engineering

**DOI:** 10.3389/fphar.2018.01329

**Published:** 2018-11-21

**Authors:** Hannah A. Strobel, Elisabet I. Qendro, Eben Alsberg, Marsha W. Rolle

**Affiliations:** ^1^Department of Biomedical Engineering, Worcester Polytechnic Institute, Worcester, MA, United States; ^2^Graduate School of Biomedical Sciences, University of Massachusetts Medical School, Worcester, MA, United States; ^3^Department of Biomedical Engineering, Case Western Reserve University, Cleveland, OH, United States

**Keywords:** drug delivery, vascular tissue engineering, vascular graft, vascular repair, drug eluting stent, nanoparticle, aneurysm

## Abstract

Cardiovascular diseases are the leading cause of death in the United States. Treatment often requires surgical interventions to re-open occluded vessels, bypass severe occlusions, or stabilize aneurysms. Despite the short-term success of such interventions, many ultimately fail due to thrombosis or restenosis (following stent placement), or incomplete healing (such as after aneurysm coil placement). Bioactive molecules capable of modulating host tissue responses and preventing these complications have been identified, but systemic delivery is often harmful or ineffective. This review discusses the use of localized bioactive molecule delivery methods to enhance the long-term success of vascular interventions, such as drug-eluting stents and aneurysm coils, as well as nanoparticles for targeted molecule delivery. Vascular grafts in particular have poor patency in small diameter, high flow applications, such as coronary artery bypass grafting (CABG). Grafts fabricated from a variety of approaches may benefit from bioactive molecule incorporation to improve patency. Tissue engineering is an especially promising approach for vascular graft fabrication that may be conducive to incorporation of drugs or growth factors. Overall, localized and targeted delivery of bioactive molecules has shown promise for improving the outcomes of vascular interventions, with technologies such as drug-eluting stents showing excellent clinical success. However, many targeted vascular drug delivery systems have yet to reach the clinic. There is still a need to better optimize bioactive molecule release kinetics and identify synergistic biomolecule combinations before the clinical impact of these technologies can be realized.

## Introduction

Every 40 seconds an American dies from cardiovascular disease, the leading cause of death in the United States. It is estimated that by 2030, 43.9% of Americans will be living with some form of cardiovascular disease (Go et al., 2014). Some diseases, such as atherosclerosis, can lead to life-threatening blood vessel occlusions. While some preventative medications exist, invasive procedures such as angioplasty, stenting, and bypass surgery are often required to restore blood vessel patency. However, these procedures provide only temporary solutions; it is estimated that up to 15–50% of angioplasties, 16–30% of saphenous vein bypass grafts, and up to 90% of synthetic coronary bypass grafts fail within 1–3 years (Lemson et al., 2000; Goldman et al., 2011; Kennealey et al., 2011; Siracuse et al., 2012; Marmagkiolis et al., 2014). Other vascular diseases such as aneurysm cannot currently be prevented or treated with medication, and must be surgically repaired.

Improvements in peripheral or coronary artery bypass grafting (CABG) in particular have made little clinical progress over the past several decades. The standard of care is to use autologous saphenous veins or internal mammary arteries as donor graft material. However, autologous vein and artery grafts are unavailable in approximately one-third of patients due to severity of vascular disease or previous surgeries (Veith et al., 1979). Synthetic grafts can be effective for surgical bypass of large diameter arteries. Coatings with molecules such as heparin have significantly improved outcomes in applications such as femoropopliteal bypass grafts (Devine et al., 2004; Walluscheck et al., 2005; Lindholt et al., 2011). However, synthetic materials still fail consistently when used as bypass grafts for high-flow, small diameter vessels, such as peripheral and coronary arteries (Pashneh-Tala et al., 2015). When used for CABG, polytetrafluoroethylene (PTFE) has a 2-year patency rate of only 32%, compared to greater than 90% for saphenous vein grafts (Chard et al., 1987; Shah et al., 2005; Hadinata et al., 2009). Thus, new alternatives may be needed for patients who lack suitable autologous grafts for bypass procedures.

Tissue engineered blood vessels (TEBVs) are being investigated as alternatives to synthetic grafts for CABG and other bypass grafting applications. TEBVs may be advantageous because they may more closely mimic the native structure and function of arteries than synthetic materials. In some cases, TEBVs may degrade and be entirely replaced by native tissue (Hibino et al., 2010). Several TEBVs have already been tested in clinical trials for hemodialysis access (Wystrychowski et al., 2014; Lawson et al., 2016) and cavopulmonary conduits (Hibino et al., 2010). While many advances have been made, TEBVs still face challenges such as incomplete endothelialization, thrombosis, stenosis following implantation, and limited function. For example, a recent clinical trial for hemodialysis access grafts reported 1-year primary patency rates of 28%, although secondary patency rates were 89% (Lawson et al., 2016). The low primary patency was primarily due to thrombosis. While this is an improvement over synthetic PTFE grafts, which have 41% primary and 59% secondary 1-year patency rates (Hodges et al., 1997), there is still room for improvement. Additionally, no TEBVs have moved forward into clinical trials specifically for CABG, as this is especially challenging due to the small diameter and high flow rate in the coronary artery. Controlled release of growth factors and other molecules directly within TEBVs may help address some of these problems.

While many different drugs have shown clinical efficacy for treating vascular disease, many are either ineffective or toxic when delivered systemically (Bea et al., 2002; Johnson et al., 2005; Marupudi et al., 2007). For example, statins, which are typically used to prevent restenosis, have also been shown to reverse existing atherosclerotic lesions, but only at doses that are toxic to humans (Bea et al., 2002; Johnson et al., 2005). Targeted delivery of these drugs using biomaterial and tissue engineering approaches may help solve this problem by allowing a much higher dose to be delivered directly to the diseased site.

In this review, we discuss how controlled release of drugs and growth factors can be used for a wide range of vascular intervention applications, from targeted treatment of vascular diseases to improving the function of TEBVs for bypass grafting. Many approaches have been developed to achieve targeted bioactive molecule delivery and localized release in the vasculature, including implantation of drug-eluting stents or bypass grafts, or delivery of specialized bioactive molecule-loaded nanoparticles designed to target sites of injury or disease.

## Vascular Disease and Intervention – Opportunities for Targeted Drug Delivery

Localized drug delivery is an ideal strategy for treating vascular diseases such as atherosclerosis, restenosis, and aneurysm, as these focal diseases affect only a small region of the blood vessel. Systemic treatments for these diseases are often ineffective, or cause harmful side effects. To address this challenge, biomaterial approaches are being developed and tested clinically for delivering therapeutics directly to sites of injury and disease.

### Atherosclerosis and Restenosis

**FIGURE 1:**
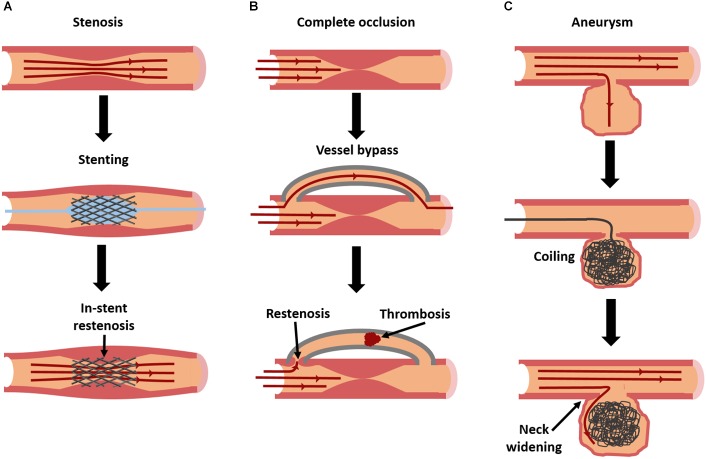
Current interventions for vascular diseases, and their modes of failure. Stenosis **(A)**, caused by atherosclerosis or intimal hyperplasia, is frequently treated with stent placement [blue balloon in **(A)** used to deploy stent] to restore patency. However, in-stent restenosis is a frequent complication. In severe cases of occlusion **(B)**, a complete vessel bypass may be necessary. With bypass grafting, there is a risk of failure due to thrombosis or restenosis at graft anastomoses. Aneurysms **(C)** can be treated with an aneurysm coil, to fill the aneurysmal sac and prevent further dilation. However, over time coils can begin to leak, allowing fluid to re-enter and further enlarge the aneurysm.

Atherosclerosis is characterized by the buildup of lipids in the vascular wall, triggered by endothelial dysfunction and inflammation. If left untreated, unstable lesions with necrotic cores may rupture and trigger a life-threatening thrombosis. Even if they do not rupture, lesions can begin to occlude the vessel and restrict blood flow to vital organs. While some preventative medications exist, treatments for large or ruptured lesions are largely invasive. A balloon angioplasty can be inflated in a partial occlusion to restore blood flow (Figure 1A; balloon shown in blue). Stents can also be put in place to mechanically hold the vessel open for a longer period (Figure 1A). In severe cases, the diseased area may need to be replaced or bypassed altogether with a vascular graft (Libby and Theroux, 2005) (Figure 1B). While effective, each of these invasive treatments can damage the endothelium, triggering a cascade of events that leads to intimal hyperplasia (restenosis), or the overgrowth of smooth muscle cells (SMCs) into the vessel lumen (Kornowski et al., 1998). Drug-eluting biomaterials may be one solution to overcoming these problems by locally delivering treatments to heal or prevent these diseases.

### Aneurysm

Aneurysms are caused by the localized degradation of the arterial elastin and elastic fibers by matrix metalloproteinases (MMPs). This causes the vascular wall to dilate and weaken, and ultimately rupture (Thompson and Parks, 1996). There are two types of aneurysms. Fusiform aneurysms affect the entire circumference of the vessel region, whereas saccular aneurysms affect only a focal region of the circumference.

There are limited options for treating aneurysms. Once they reach a critical size, surgical intervention becomes necessary. For fusiform aneurysms, the affected region is either replaced or reinforced with a synthetic vascular graft (Tarafdar and Gannon, 2017). For smaller saccular aneurysms in regions such as the brain, the dilated region can be surgically “clipped,” or tied off, so blood cannot flow into the dilated region, although this procedure is highly invasive. In some cases, “flow diverters” are used, which are specially designed stents that block blood from entering the aneurysm. Alternatively, a metallic coil can be guided up through the vasculature to the aneurysm, and essentially “stuffed” in to fill the dilation and prevent blood from entering (Pierot and Wakhloo, 2013) (Figure 1C).

### Drug Delivery Opportunities for Vascular Intervention

**FIGURE 2:**
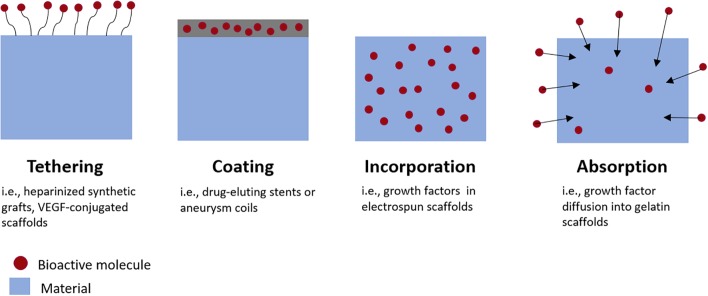
Methods for incorporating bioactive molecules into medical devices or tissue engineered grafts. Growth factors can be tethered to graft surfaces, incorporated directly into material coatings, incorporated directly within scaffold materials during fabrication, or absorbed into the material post-fabrication.

There are numerous methods for incorporating drugs or growth factors into biomaterials, summarized schematically in Figure 2. Molecules such as heparin can be immobilized on the material surface, an approach which is frequently utilized for reducing thrombosis risk on synthetic vascular grafts (Biran and Pond, 2017). Growth factors can also be bound to heparin-coated surfaces. This strategy results in a longer, sustained release of growth factors than binding them to non-heparinized surfaces (Jeon et al., 2007).

Alternatively, therapeutics can be encapsulated within biomaterials during fabrication. For example, with emulsion electrospinning, growth factors are combined with a polymer solution and then electrospun into a scaffold with growth factor-encapsulating fibers (Zhang et al., 2013). Depending on the biomaterial used, molecules encapsulated by materials may be released by affinity interactions, diffusion, bulk or surface degradation of the material; or a combination of release mechanisms. This is advantageous because it enables sustained tunable release of the molecule with minimal burst release (Lee et al., 2007). Burst releases occur when a large portion of the molecules are released in a very short period of time, which can have toxic or other off-target effects on cells depending on the molecule released.

Molecules can also be loaded via diffusion into materials after fabrication, by soaking the material in a bioactive factor solution. For example, growth factors can diffuse into gelatin scaffolds and bind electrostatically to the gelatin. The growth factor is then released by dissociation and diffusion as the gelatin proteolytically degrades (Tabata and Ikada, 1998). Growth factor loading and release methods are reviewed in greater detail in Lee et al. (2011).

Each of these loading techniques results in materials with different bioactive factor release kinetics. Different types of release kinetics may be ideal for different applications. This wide range of available systems and achievable release kinetics may enable drug-delivery systems to be customized for many applications in tissue engineering and regenerative medicine.

### Device Design Criteria

When designing drug-eluting biomaterials for tissue engineering or regenerative medicine, many factors need to be considered. It is critical that the material used is biocompatible and will not trigger any local or systemic toxic effects. Many drugs are released as material degrades, and polymer degradation products can be harmful to cells (Higgins et al., 2003). Consistency in the manufacturing process is also important; uneven drug loading may cause too much or too little drug to be delivered. The material fabrication process also plays a role in the effectiveness of a therapeutic. Growth factors, nucleic acids, and many drugs may denature and/or degrade upon heating, so if a heating step is required in the manufacturing process, the bioactivity of the molecules may be diminished. Any material intended for cell culture or implantation must be sterilized without degrading, and may need to be stored for extended periods of time. In some cases, it may be possible to load the material with a drug directly before use. However, this is not always an option, especially for treatments such as stent placement or angioplasty, where time is often a critical factor. Thus, an ideal drug-loaded biomaterial is stable enough for long-term storage and can be available off-the-shelf.

More specific design criteria varies considerably depending on the disease, target mechanism, delivery mechanism, and molecule being delivered. Thus, it is challenging to create broad specifications for drug-eluting biomaterial systems. The delivery mechanism must be designed in such a way that the molecule can reach its intended target. For example, a therapeutic that inhibits SMC proliferation, such as paclitaxel, must be able to penetrate the endothelium to reach the medial layer of the blood vessel. However, a nanoparticle that targets the endothelium, such as vascular endothelial growth factor (VEGF), may not need to penetrate beyond the luminal surface of the blood vessel. Different molecules may require different doses or release kinetics to be effective, depending on how long they remain in the tissue. For example, paclitaxel accumulates and remains in tissue for longer periods of time than sirolimus, and thus does not require as prolonged of a release (discussed further below) (Levin et al., 2004).

## Localized Drug Delivery from Vascular Stents

Stent placement has been used to restore blood flow following partial vessel occlusions since the 1980s. However, the original bare-metal stents can trigger life threatening conditions such as thrombosis, and have high rates of restenosis (Chen et al., 2006). Drug-eluting stents can provide localized delivery of therapeutics directly to the blood vessel wall and prevent in-stent restenosis. When designing drug-eluting stents, many factors need to be considered. Optimizing the release kinetics is critical for maximizing drug effectiveness and minimizing adverse effects. Release profile and drug distribution within the tissue can be affected by a number of factors, including the properties of the drug (Bozsak et al., 2015), stent or coating material and drug release mechanism (Acharya and Park, 2006), stent geometry (Seo et al., 2016), coating thickness, stent-induced changes in blood flow patterns (Seo et al., 2016), initial drug loading concentration (Bozsak et al., 2015), presence of thrombi in and around the stent (Hwang et al., 2005), and drug absorption capacity of the surrounding tissue (Balakrishnan et al., 2007). Therapeutics discussed in this section are summarized in Table 1.

**Table 1:** Molecules delivered for atherosclerosis treatment.

Molecule	Delivery mechanism	Effects
Sirolimus	Drug eluting stents	Inhibit cellular proliferation and migration
Paclitaxel	Drug eluting stents, Nanoparticles	Inhibit cellular proliferation and migration
Novolimus	Bioresorbable vascular scaffolds	Inhibit cellular proliferation and migration
Everolimus	Bioresorbable vascular scaffolds	Inhibit cellular proliferation and migration
Amphilimus	Nanoparticles in drug eluting stents	Inhibit cellular proliferation and migration
Biolimus	Drug eluting stents	Inhibit cellular proliferation and migration
Heparin	Drug eluting stents, Synthetic vascular grafts	Prevent thrombosis
CD34	TEBVs, Drug eluting stents	Accelerate endothelialization
VEGF	Drug eluting stents	Accelerate endothelialization
Simvastatin	Nanoparticles	Inhibit SMC proliferation and migration, promote endothelial health, anti-inflammatory, lower cholesterol
IL-10	Nanoparticles	Anti-inflammatory
Prednisolone	Nanoparticles	Anti-inflammatory
Ac2-26	Nanoparticles	Anti-inflammatory
Carmustine	Nanoparticles	Inhibit cellular proliferation
VEGF plasmids	Nanoparticles	Increase VEGF production to heal damaged endothelium
SiRNA, microRNA	Drug eluting stents, nanoparticles	Enhance endothelial function, prevent macrophage accumulation, reduce adhesion molecule receptors, suppress SMC proliferation

*Summary of bioactive factors discussed in Sections “Localized Drug Delivery From Vascular Stents” and “Nanoparticle-Mediated Drug Delivery for Restenosis Prevention,” their delivery mechanisms, and effects*.

### Paclitaxel and Sirolimus-Eluting Stents

The two original drug-eluting stents, Cypher and Taxus, were fabricated from stainless-steel scaffolds with a permanent polymer coating designed to release sirolimus (Cypher) or paclitaxel (Taxus). Both of these drugs are known to prevent SMC proliferation and migration (Marx et al., 1995; Axel et al., 1997). The Cypher and Taxus stents were approved by the FDA based on their ability to reduce restenosis compared to bare metal stents (Sousa et al., 2001; Grube et al., 2002; Morice et al., 2002; Colombo et al., 2003; Moses et al., 2003; Stone et al., 2004). However, reduced endothelialization and delayed healing compared to bare-metal stents led to high incidences of late in-stent thrombosis. This may be attributed to drug-mediated inhibition of endothelial cell (EC) proliferation, in addition to drug effects on target SMCs, and localized inflammation (Farb et al., 2001; Joner et al., 2006; Pfisterer et al., 2006; Finn et al., 2007a,b; Nakazawa et al., 2008). Sensitivities to the materials used may also play a role in prolonged inflammation (Suzuki et al., 2001).

Paclitaxel and sirolimus have different transport dynamics through the arterial wall, leading paclitaxel to accumulate in the adventitia, rather than the media (Levin et al., 2004). Additionally, modeling simulations indicate that paclitaxel unbinds from tissue 20 times slower than sirolimus, thus causing it to remain in the arterial wall for a longer duration than sirolimus (Balakrishnan et al., 2007; Bozsak et al., 2014; Bozsak et al., 2015). Because the Taxus stent delivers a relatively high dose of paclitaxel over 30 days, it may accumulate in very high levels in the arterial wall and trigger localized inflammation (Radeleff et al., 2010; Bozsak et al., 2015). This may be one possible explanation for why paclitaxel-eluting stents are less effective than sirolimus-eluting stents (Dibra et al., 2005; Kastrati et al., 2005). It is possible that decreasing the loading concentration of paclitaxel in stents and releasing either one short burst, or a much slower release over a period of years, may reduce buildup in the arterial wall, and prevent SMC hyperplasia without delaying the healing process (Bozsak et al., 2014; Bozsak et al., 2015).

The idea of designing stents with a large initial burst release has been applied to paclitaxel-eluting angioplasty balloons. These balloons deliver a short burst of targeted therapy to a diseased region, which accumulates and remains in the arterial wall for an extended period. Paclitaxel-eluting balloons have reduced restenosis rates compared to traditional angioplasty balloons (Rittger et al., 2012; Byrne et al., 2013; Miura et al., 2017). While paclitaxel-eluting stents have largely been phased out, it is possible that further optimization of their release kinetics may have improved clinical results. This highlights the importance of understanding and tuning the release kinetics and diffusion properties of therapeutics released by stents and other systems for each specific drug and clinical indication.

### Second Generation Drug-Eluting Stents

Second-generation drug-eluting stents used similar designs to Taxus and Cypher, but with improved drug release kinetics, more biocompatible materials, and altered stent geometry. There are numerous commercially available variations of these designs, most of which elute sirolimus derivatives. As a result of these changes, clinical outcomes have improved compared to first generation designs, especially in improving stent safety, and this has led second generation drug-eluting stents to become the current gold standard (Navarese et al., 2013; Valgimigli et al., 2014; Kobayashi et al., 2016). However, others have suggested that the improvements have not reduced the overall risk of late in-stent thrombosis, indicating that a different approach to stent design is needed (Tada et al., 2013). Second generation drug-eluting stents are reviewed in detail in Ho et al. (2016) and Akinapelli et al. (2017).

### Other Stent Designs

Despite the success of second-generation drug-eluting stents, restenosis and thrombosis remain a problem, and currently available drug-eluting stents may not be appropriate for all patients. Thus, other approaches to stent design are being developed. Immobilized heparin on the stent surface may be an additional option for preventing thrombosis. There is currently one commercially available heparin-eluting stent, the Viabahn stent, which is fabricated from a nitinol base with a heparin-bound ePTFE coating. The Viabahn stent demonstrated improved patency rates in clinical trials compared to bare-metal stents (Lammer et al., 2013; Saxon et al., 2013).

Polymer-free drug-eluting stents may be advantageous for patients with polymer sensitivities. While loading drugs onto metallic surface can be challenging, several different strategies have proven effective. For example, the Cre8 stent releases nanoparticles containing amphilimus from reservoirs on the abluminal side of the stent (Carrie et al., 2012). The BioFreedom stent adheres biolimus A9 to a micro-structured metallic surface (Urban et al., 2015). The VESTAsync stent has sirolimus loaded into a microporous hydroxyapatite coating (Costa et al., 2009; van der Giessen et al., 2009). These techniques have had comparable clinical results to second-generation drug-eluting stents, and may reduce the risk of delayed healing (Costa et al., 2009; Carrie et al., 2012; Urban et al., 2015).

More complex stent designs have focused on further optimizing drug release kinetics, in addition to material and mechanical properties. Stents containing reservoirs can be filled with drugs that are released through small holes on the abluminal side of the stent only, allowing for more targeted drug delivery over a longer period (Finkelstein et al., 2003; Krucoff et al., 2008). Other groups have tried using a layer-by-layer assembly approach to coating stents, with materials such as chitosan or hyaluronic acid and therapeutics such as heparin and growth factors, to further control and customize drug release kinetics (Meng et al., 2009; Hossfeld et al., 2013; Liu et al., 2013; Su et al., 2013). Layer-by layer assembly can allow for the release of multiple drugs, which may be more effective than single-drug approaches. For example, coatings releasing sirolimus and heparin have been tested, which act to prevent restenosis and thrombosis, respectively (Su et al., 2013). Heparin also has a high affinity for growth factors, which become immobilized on its surface. In Liu et al. (2013), heparin was immobilized to the stent surface using an avidin–biotin system, and then CD34 and VEGF are bound to the heparin, with the goal of accelerating endothelialization (Liu et al., 2013). Both of these studies have shown promise *in vitro*.

### Bioresorbable Vascular Scaffolds

More recent studies are focused on creating bioresorbable drug-eluting stents, or vascular scaffolds, from either metallic or polymeric materials. By completely degrading, these bioresorbable vascular scaffolds may alleviate many of the negative effects observed with metallic polymer-coated drug-eluting stents. They may also allow for the restoration of normal function and vasomotion of the vessel (Serruys et al., 2016). With non-degradable stents, a permanent focal decrease in vascular compliance can cause regions of compliance mismatch, increasing the risk of restenosis (Farhan et al., 2009; Selvarasu et al., 2011).

The two most widely used polymeric bioresorbable vascular scaffolds, the DESolve and Absorb bioresorbable vascular scaffolds, are both fabricated from poly-L-lactic acid (PLLA), and elute novolimus and everolimus, respectively. The DESolve bioresorbable vascular scaffold degrades completely in about 1 year, while the Absorb bioresorbable vascular scaffold degrades in approximately 3 years. Initial clinical trials of both bioresorbable vascular scaffolds were promising (Abizaid et al., 2016; Serruys et al., 2016). However, recent studies suggested the overall risk of death is similar between second generation drug-eluting stents and bioresorbable vascular scaffolds (Pandya et al., 2016; Stone et al., 2016; El-Hayek et al., 2017), and the risk of very late thrombosis (1–2 years after implantation) may even be higher than in drug-eluting stents (Toyota et al., 2017). Still, more time is needed to determine the extent of other potential long-term benefits, such as restored vessel function.

In addition to degradable polymers, degradable metals can also be used for bioresorbable vascular scaffolds. The DREAMS 2G bioresorbable vascular scaffolds is fabricated from a magnesium alloy with a sirolimus-loaded PLA coating. When the magnesium alloy degrades, it is first converted into hydrated magnesium oxide, and then into magnesium phosphate. Then it is replaced by amorphous calcium phosphate, which remains in the tissue. The magnesium diffuses out of the tissue and is absorbed by the body. The entire degradation process takes about 1 year (Haude et al., 2016). In clinical trials, the DREAMS 2G bioresorbable vascular scaffold performed similarly to polymeric bioresorbable vascular scaffolds, although long-term studies and direct comparisons are still needed (Haude et al., 2016). Bioresorbable vascular scaffolds are reviewed in detail in Bowen et al. (2016) and Ho et al. (2016).

### Future of Stent Design

While release kinetics are critical to the performance of drug-eluting stents, they are extremely challenging to measure *in vivo*. This makes it difficult to engineer stents with specific release profiles, and several design iterations may be needed to achieve the desired outcome. Recent mathematical models combining *in vitro* with *in vivo* studies have increased our understanding of therapeutic drug release from stents, which may help reduce the number of iterations needed for success (McGinty et al., 2013, 2017; Bozsak et al., 2014, 2015). In the future, these models may be key to fabricating stents that deliver therapeutics at doses that inhibit SMC proliferation and prevent restenosis, but without toxic effects on ECs that have resulted in delayed healing, inhibition of re-endothelialization, and late thrombosis.

Many new molecules also hold promise for improving future drug-eluting stent designs. The possibility of delivering small-interfering ribonucleic acid (siRNA delivery) is currently being investigated (Hossfeld et al., 2013; Che et al., 2016; Cho and Park, 2017). siRNA are short double-stranded RNA molecules that interfere with the expression of specific genes. Stents have incorporated siRNA with the goal of reducing adhesion molecule receptors to reduce thrombosis and inflammation (Hossfeld et al., 2013), or suppressing SMC proliferation and preventing restenosis (Che et al., 2016). Gene-eluting stents are also being explored, as targeted gene therapy may upregulate production of growth factors or other molecules that may reduce intimal hyperplasia and thrombosis [reviewed in detail in (Yin et al., 2014) and (Adeel and Sharif, 2016)].

As a wider range of stent materials and therapeutics become available with different dosage kinetics, stent selection may be tailored to an individual patient’s needs, in order to deliver the most beneficial dosage of a specific therapeutic(s) to the diseased location for a precise duration.

## Nanoparticle-Mediated Drug Delivery for Restenosis Prevention

For minor atherosclerotic lesions, targeted drug delivery via nanoparticles may be a less invasive option than stents or vascular grafts. Nanoparticles have been used clinically for targeted drug delivery to cancerous tumors [reviewed in (Brannon-Peppas and Blanchette, 2012)]. For atherosclerosis, many nanoparticles are in clinical trials to aid in imaging and diagnosing lesions. These specialized nanoparticles may be visible with imaging techniques such as MRI, and others may deliver contrast agents directly to the diseased site [reviewed in (Palekar et al., 2015)]. Here, we will focus on nanoparticles that are in development for the delivery of therapeutics to heal atherosclerotic lesions or prevent their progression.

### Nanoparticle Design

The success of any nanoparticle-mediated treatment is determined by the nanoparticles’ ability to reach their target, typically following intravenous injection, and to provide the optimal dose of drug over a sustained period. These qualities are determined by the nanoparticle size (Walkey et al., 2012; Tan et al., 2013), surface properties (Walkey et al., 2012), particle geometry (Tan et al., 2013), shear stress and flow rate in the blood vessel (Klingberg et al., 2015), and drug release kinetics (Panyam and Labhasetwar, 2004). Many different materials have been used for fabricating drug-eluting nanoparticles, including synthetic polymers [reviewed in (Wang et al., 2016)], lipoproteins [reviewed in (Damiano et al., 2013; Harisa and Alanazi, 2014)], lipids (Shiozaki et al., 2016), and metals (Weakley et al., 2011). Different materials and design criteria may be needed depending on the type of drug to be released, and the intended target of the nanoparticle.

**FIGURE 3:**
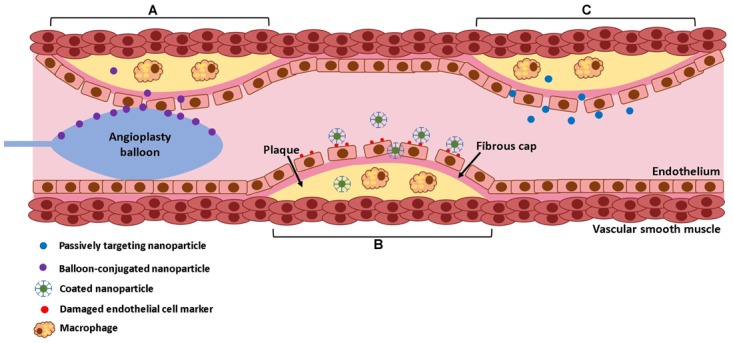
Mechanisms of nanoparticle delivery. Nanoparticles can be delivered directly to the lesion site by balloon angioplasty **(A)**, they can be targeted using conjugated antibodies that target specific surface markers on the lesion **(B)**, or they can passively diffuse into the lesion due to increased endothelial permeability **(C)**.

In addition to material considerations, the nanoparticle targeting mechanism must be considered (Figure 3). After injection, nanoparticles face several barriers to reaching their target. They may be uptaken by macrophages, distribution may be limited by blood flow, pressure gradients, or cellular internalization [reviewed in (Blanco et al., 2015)]. Thus, developing an effective targeting mechanism may increase nanoparticles’ ability to reach their intended target. Direct application via balloon angioplasty can be used, although intravenous injection is more common. Nanoparticles may naturally accumulate in atherosclerotic lesions, due to increased endothelial permeability at the diseased site, a process known as passive targeting (Duivenvoorden et al., 2014). This is the most common approach for nanoparticles targeting atherosclerotic lesions. Measuring the exact percent targeting efficiency of nanoparticles *in vivo* is challenging, as nanoparticles rapidly distribute throughout the body. Still, it is often possible to use labeled nanoparticles to measure relative concentration in select organs. For example, van der Valk et al. (2015) reported that 75% of plaque macrophages contained nanoparticles after IV injection with passive targeting. Still, it is unknown what fraction of injected nanoparticles remain in the body. Some materials can alternatively be taken up by macrophages or monocytes in the bloodstream, which may then accumulate in atherosclerotic plaque (Katsuki et al., 2014). Others may be magnetically guided to the diseased site (Chorny et al., 2009). Alternatively, active targeting may be used, where nanoparticles are conjugated with antibodies targeting specific proteins that are overexpressed at sites of vascular injury, such as collagen IV (Fredman et al., 2015), E-selectin (Ma et al., 2016), vascular cell adhesion molecule 1 (VCAM-1) (Nahrendorf et al., 2006), CD36 (Nie et al., 2015), and α*_v_*β_3_ integrin (Winter et al., 2008). These active targeting systems may improve nanoparticles ability to reach their target. For example, the collagen IV targeting system described in Fredman et al. (2015) compared nanoparticle accumulation in the liver, spleen, and aorta, and found that 70% of measured particles were in the aorta. In addition, nanoparticles activated by elevated shear stresses have been developed. Regions of luminal narrowing caused by atherosclerosis, restenosis, or thrombosis experience elevated levels of shear. Thus, nanoparticles that release therapeutics when exposed to high shear stresses may be effective for targeting atherosclerotic lesions or intimal hyperplasia (Korin et al., 2012). Nanoparticle design is further reviewed in Blanco et al. (2015).

### Therapeutics for Nanoparticle-Mediated Delivery

Since atherosclerosis is a very complex disease, combinations of drugs or growth factors may be required for effective treatment. Therapeutics targeting inflammatory cells, SMC proliferation, thrombosis, and dysfunctional endothelial cells have all shown promise in pre-clinical or clinical studies. HMG Co-A reductase inhibitors (known as statins) are widely used for systemic prevention of atherosclerosis, due to their pleiotropic effects on cholesterol levels, SMC proliferation, inhibition of inflammation, and promotion of endothelial health (Liao, 2005). Statins have also been shown to stabilize and reverse advanced atherosclerotic plaques in animals, but only at high doses that are not approved for humans (Bea et al., 2002; Johnson et al., 2005). Thus, the targeted delivery of higher doses via nanoparticles may be able to reverse lesion progression without the harmful effects of systemic delivery (Winter et al., 2008; Zago et al., 2013; Duivenvoorden et al., 2014; Katsuki et al., 2014; Alaarg et al., 2017). A recent study compared the effects of simvastatin delivery via intravenously injected nanoparticles fabricated from Polyethylene glycol (PEG)-ylated polymeric micelles, high-density lipoproteins (HDL), and PEGylated lysosomes on advanced atherosclerotic lesions, with the goal of decreasing inflammation by reducing macrophage burden (Alaarg et al., 2017). They found that PEGylated polymeric micelles yielded greater reductions in macrophage burden in advanced atherosclerotic plaques. This may be due to their slower release of simvastatin and better ability to target macrophages than the other nanoparticle types. This highlights the importance of optimizing nanoparticle design and release kinetics for maximum drug effectiveness.

Other molecules that target inflammation have also shown promise in pre-clinical studies, including interleukin 10 (IL-10) (Kamaly et al., 2016), steroids (Lobatto et al., 2015), and the proresolving peptide Ac2-26, a protein that mimics the effects of annexin 1 (Fredman et al., 2015). van der Valk et al. (2015) tested IV injected, passive targeting, prednisolone-loaded liposomal nanoparticles in a clinical study. While the nanoparticles had shown promise in previous rabbit models (Lobatto et al., 2015), they did not reduce inflammation in human lesions (van der Valk et al., 2015). This may be due to the short-term (10 days) nature of the study. The authors were also unable to verify that the nanoparticles were accumulating in lesions. In the future, nanoparticles that can be imaged may enable lesions to be both visualized and treated at the same time, and alleviate uncertainty over whether they are reaching their target. Additionally, the lack of observed benefit in clinical studies may be due to differences in atherosclerotic plaque formation in animals compared to humans. Rabbits, like many small animals, will not form plaque without a high-cholesterol diet. This results in much higher cholesterol levels than are typically seen in humans, and lesions that are more fatty and inflammatory that human atherosclerotic plaques (Cullen et al., 2003; Yanni, 2004). Mice are also commonly used, but require genetic manipulation in addition to diet changes to develop plaque lesions (Calara et al., 2001). Because small animals have similarly sized cells to humans but much smaller blood vessels, there are fewer cells involved in vessel function and plaque formation, which may affect disease progression. These animals also have considerable genetic homogeneity compared to humans, making it challenging to replicate the large patient-to-patient variability typically seen in humans (Cullen et al., 2003).

Chemotherapeutic agents have been studied as potential atherosclerosis treatments due to their potent anti-proliferative effects. Intravenously injected lipid nanoparticles loaded with the drug carmustine successfully decreased lesion area by reducing SMC proliferation, secretion of inflammatory factors, and macrophage burden in rabbits (Daminelli et al., 2016). Shiozaki et al. (2016) recently published a pilot clinical study where cholesterol-rich non-protein nano-emulsion (LDE) particles, which resemble low-density lipoproteins, were loaded with paclitaxel oleate. When delivered systemically via intravenous injection, the nanoparticles were able to reduce total plaque in patients, though the difference was not significant (Shiozaki et al., 2016).

Due of the complexity of atherosclerosis, the release of multiple factors with different targets may provide a greater benefit than one factor alone. Zhu et al. (2017) designed bilayered poly(lactide-co-glycolide)/poly(vinyl alcohol) (PLGA/PVA) nanoparticles loaded with VEGF-encoding plasmid DNA and paclitaxel. Plasmid DNA encoding genes for growth factors and other proteins can enable upregulated production of a particular molecule without additional stimuli. This system is designed to first increase VEGF production and heal the endothelial layer, and then inhibit SMC proliferation. Results were promising in a rabbit model, where histological analysis showed endothelialization and decreased stenosis after 28 days in animals treated with the bilayered nanoparticles, delivered via angioplasty balloon (Zhu et al., 2017). Using plasmids to induce growth factor production in cells may also help address concerns over growth factor stability and reduce the risk of potentially toxic growth factor burst releases. SiRNA and microRNA are also potential therapeutics for treating atherosclerosis. Ma et al. (2016) utilized intravenously injected polymer nanoparticles to deliver microRNA to enhance endothelial function and reduce lesion size in a mouse model. Others have tested nanoparticle-mediated siRNA delivery, with some success (Li et al., 2010; Leuschner et al., 2011). For example, Leuschner et al. (2011) used siRNA to prevent macrophage accumulation in atherosclerotic plaques.

Overall, nanoparticles have great potential for targeted delivery of therapeutics to atherosclerotic lesions. A wide range of materials, therapeutics, and molecular targets have been investigated, although very few have advanced to clinical trials. Therapeutics and nanoparticle properties discussed in this section are summarized in Tables 2 and 3. With such a complex disease, it is likely that multi-bioactive factor approaches will be needed to effectively heal atherosclerotic lesions, as single bioactive factor approaches may be ineffective. Moving forward, issues such as consistency of nanoparticle fabrication, optimization of nanoparticle targeting and bioactive molecule release, premature nanoparticle clearance from the body, and the potentially harmful effects of some nanoparticles must also be addressed before these treatments become clinically available.

**Table 2:** Molecules delivered for aneurysm repair.

Molecule	Delivery mechanism	Effects
VEGF	Aneurysm coil	Enhance clot organization, encourage endothelium formation
FGF	Aneurysm coil	Promote wound healing
SEK-100	Aneurysm coil	Accelerate healing
Tenascin-C	Aneurysm coil	Accelerate healing
SDF-1α	Aneurysm coil	Accelerate healing
Doxycycline	Nanoparticles	Prevent elastin degradation
TGF-β1	Nanoparticles	Increase elastin synthesis
Hyaluronan oligomers	Nanoparticles	Increase elastin synthesis
Tissue plasminogen activator	Nanoparticles	Fibrinolysis of clots

*Summary of molecules discussed in Section “Targeted Bioactive Molecule Delivery for Aneurysm Repair,” their delivery mechanism, and effect on aneurysm repair*.

**Table 3:** Nanoparticle properties.

Material	Average diameter	Molecule delivered	Targeting mechanism	Comments	Reference
Polyethylene glycol (PEG)-ylated polymeric micelles	80 nm	Simvastatin	Passive targeting	Greater reduction in plaque macrophage burden than HDL and PEGylated lysosomes in mice	Alaarg et al., 2017
PLA and PLGA with outer peptide sequence with collagen IV affinity	120 nm	IL-10	Target exposed collagen IV	Increased fibrous cap thickness, decreased necrotic cores to prevent vulnerable plaque formation in mice	Kamaly et al., 2016
PEG-modified liposomes	100 ± 10 nm	Prednisolone phosphate	Passive targeting	Anti-inflammatory effects in rabbit atherosclerotic lesions	Lobatto et al., 2015; van der Valk et al., 2015
PLGA/PEG blend	<100 nm	Ac2-26	Target exposed collagen IV	Increased lesion fibrous cap formation, decreased necrotic cores, and suppressed oxidative stress in mice	Fredman et al., 2015
Lipids resembling LDL (LDE)	60 nm	Carmustine	Bind to LDL receptors	Reduced lesion size by 90% in rabbits	Daminelli et al., 2016
LDE	52 nm	Paclitaxel oleate	Bind to LDL receptors	No toxicities detected, appeared to reduce lesion size in a small group of human patients	Shiozaki et al., 2016
PLGA/PVA	306.53 ± 16.16 nm	VEGF-encoding plasmid and paclitaxel	Locally delivered by balloon angioplasty	Increased re-endothelialization and reduced restenosis in rabbits	Zhu et al., 2017
PEG/PEI	N/A	MicroRNA	Loaded into microparticles that selectively bind to E-selectin on inflamed endothelium	miR-146a and miR181b decreased plaque size and macrophage infiltration in mice	Ma et al., 2016
Lysine with oleic acid surface modification (HB-OLD7)	>200 nm	SiRNA	Locally delivered via balloon angioplasty	Knockdown of NADPH oxidase gene by nanoparticle-mediated SiRNA delivery reduced restenosis in rats	Li et al., 2010
C12-200 lipid, distearoylphosph-atidyl choline, cholesterol, PEG-DMG formulation	70–80 nm	SiRNA	N/A	Prevents monocyte accumulate in plaque	Leuschner et al., 2011

*Summary of nanoparticles discussed in Section “Nanoparticle-Mediated Drug Delivery for Restenosis Prevention,” that have shown success in vivo and their properties*.

## Targeted Bioactive Molecule Delivery for Aneurysm Repair

### Bioactive Factor-Eluting Aneurysm Coils

One treatment method for saccular aneurysms is to “clip” or tie off the aneurysm, to prevent blood from flowing into the aneurysm to cause further dilation or bursting. Aneurysm coiling, where a coil is inserted through the vasculature and into the dilation, is often preferred over clipping because it is a much less invasive procedure, and some aneurysms in the brain may not be accessible for clipping. However, there is a risk that over time blood may leak back into the dilation, causing a secondary aneurysm. To address this, bioactive coils fabricated from degradable polymers, such as PLGA, have been developed. As bioactive polymers degrade, they trigger an inflammatory response and tissue remodeling, which causes new tissue to fill in and “heal” the aneurysm. This approach successfully reduced the rate of residual aneurysms, although it did not improve the overall rate of late aneurysm rupture or reintervention (Broeders et al., 2016).

Bioactive factor-eluting coils may be able to further enhance tissue healing. Growth factors such as VEGF may enhance clot organization and encourage endothelium formation across the aneurysm neck, preventing leakage into the coiled region (Abrahams et al., 2001; Ohyama et al., 2004). Fibroblast growth factor (FGF) is also well-established to promote wound healing and new tissue formation. Coils coated with VEGF or FGF have shown some success in animal studies (Abrahams et al., 2001; Matsumoto et al., 2003; Ohyama et al., 2004; Tsumoto et al., 2007). However, issues such as growth factor instability and suboptimal release kinetics prevented these systems from moving forward to clinical trials.

More recent approaches focus on developing more stable growth factor delivery systems. Wang et al. (2014) tested a coil coated with VEGF-loaded poly(D,L-lactide)-7co-(1,3-trimethylene carbonate), and achieved a sustained VEGF release curve over 25 days (Wang et al., 2014). However, it should be noted that *in vitro* release experiments do not account for the many complexities of the *in vivo* environment, including proteases, the inflammatory response, and other factors that may affect material degradation and molecule release rates. Coils coated with more stable molecules such as SEK-1005, a TGF-β inducing peptide known to promote wound healing, have also been studied. While this system accelerated aneurysm healing in rats, inconsistent growth factor loading was a problem (Sano et al., 2010). Hamada et al. (2014) developed a platinum coil with tenascin-C, a protein known to promote fibrosis, loaded in a gellan sulfate core. Gellan sulfate is a heparin-like molecule that strongly binds to tenascin-c, resulting in a slow, controlled release. Additionally, because the protein is released from the core, it avoids potentially harmful buildup at the coil-arterial interface (Toma et al., 2005; Miura et al., 2016). Pre-clinical trials showed improved tissue formation over the aneurysm neck and improved intra-aneurysmal clot organization, which may ultimately lead to improved tissue remodeling (Hamada et al., 2014; Miura et al., 2016). Recently, Gao et al. (2014) designed a coil with a silk-fibroin coating that eluted stromal-cell derived factor 1α (SDF-1α), which demonstrated a steady release of growth factor over 21 days as the coating degraded. In preliminary rat studies, they observed improved tissue growth within the aneurysmal region compared to uncoated coils (Gao et al., 2014).

While many different drugs and growth factors have potential for healing aneurysms, designing coils with optimal release kinetics, as with drug-eluting stents, is key. Delivering too much of a molecule in a short period of time, or not delivering enough, may have negative consequences. Thus, developing systems that can provide controlled, sustained release of stable therapeutics for enhancing clot organization and tissue growth is critical.

### Nanoparticle Targeting for Aneurysm Repair

Unlike molecules delivered on aneurysm coils, which aim to “fill” existing saccular aneurysms with new tissue, MMP inhibitors may be effective at preventing elastin degradation and halting aneurysm growth. Specifically, doxycycline has been shown to reduce plasma MMP levels and inflammation of the vessel wall (Baxter et al., 2002; Lindeman et al., 2009). However, systemic delivery causes dose-dependent side effects (Baxter et al., 2002). The Ramamurthi group has investigated localized delivery of the MMP inhibitor doxycycline via PLGA nanoparticles. Nanoparticles were functionalized with a cationic amphiphile, which improved arterial uptake and enhanced MMP inhibitory activity (Sivaraman and Ramamurthi, 2013). They also tested nanoparticles loaded with smaller superparamagnetic iron oxide nanoparticles, in addition to doxycycline, to magnetically guide nanoparticles to the affected area (Sivaraman et al., 2017). So far, results from the above studies have shown promise in *in vitro* and *ex vivo* studies. Nosoudi et al. (2015) fabricated poly(D,L-lactide) nanoparticles for the delivery of the MMP inhibitor batimastat. Nanoparticles were conjugated with an elastin antibody that binds to degrading elastin, which may allow systemically delivered nanoparticles to target locations of aneurysm. The approach has shown promise in rats, where the intravenously injected nanoparticles were able to prevent aneurysm expansion (Nosoudi et al., 2015). In addition to MMP inhibitors, nanoparticle-mediated delivery of hyaluronan-oligomers and of transforming growth factor β1 (TGF-β1) have also shown promise as therapeutics for increasing elastin synthesis *in vitro* (Sylvester et al., 2013; Venkataraman et al., 2016).

While the aforementioned systems are promising for targeting aneurysms, bioactive molecule delivery to the vascular wall may still be hindered by the presence of clots on the aneurysm intraluminal surface. Combining these approaches with other molecules such as tissue plasminogen activator, which allows for controlled fibrinolysis of clots, may enhance their effectiveness. Nanoparticle-mediated tissue plasminogen activator delivery has shown promise *in vitro* for this purpose (Sivaraman et al., 2016). Ultimately, a more comprehensive approach where nanoparticles can first carefully dissolve clots in the aneurysm, and then deliver MMP inhibitors to stabilize the aneurysm, may be necessary.

## Targeted Delivery in Vascular Grafts

### Non-degradable Synthetic Vascular Grafts

In severe cases of vascular disease, the affected area may need to be replaced altogether with a bypass graft. Permanent synthetic grafts have been widely used for replacing large diameter blood vessels, in cases of both stenosis and aneurysm. They have excellent mechanical strength and are relatively simple and cost effective to produce on a large scale. However, in small diameter (<6 mm diameter) vessels, synthetic grafts have an extremely high risk of thrombosis and restenosis (Chard et al., 1987; Shah et al., 2005; Hadinata et al., 2009). This may be due to incomplete endothelialization, which is crucial for reducing the risk of these complications (Allen et al., 1984). The surface properties of commonly used synthetic materials can make these grafts challenging to endothelialize (Moby et al., 2007). Incorporating heparin or growth factors onto graft surfaces may help address this challenge.

### Heparinized Synthetic Grafts

**FIGURE 4:**
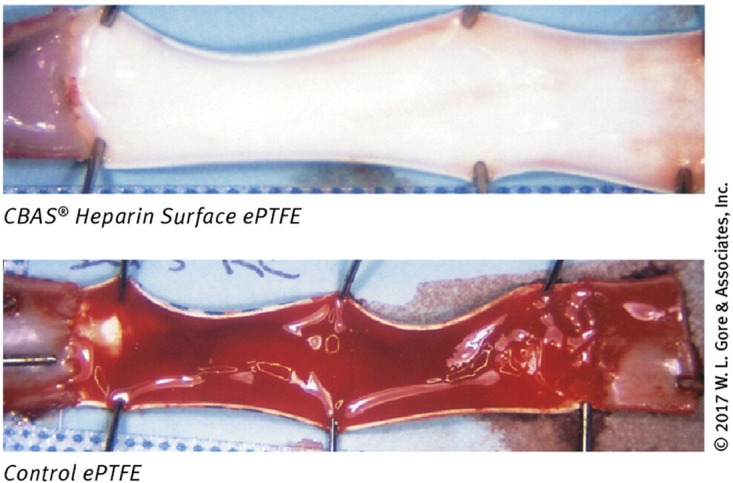
Effect of heparin on synthetic graft thrombosis. A GORE ePTFE vascular graft with or without a CBAS heparin surface coating, following a 2-h implantation in a canine carotid model. Thrombosis is clearly visible in the graft without the CBAS heparin surface. Figure reprinted from (Biran and Pond, 2017) with permission from W. L. Gore and Associates.

Synthetic grafts with incorporated heparin have been tested as early as the 1980s (Noishiki et al., 1986; Nojiri et al., 1987). Exogenous heparin is well known to inhibit SMC proliferation, prevent thrombosis, and encourage endothelialization, though high systemic doses can be harmful (O’Donnell, 2012). Thus, targeted delivery of heparin by immobilization on synthetic grafts, particularly those fabricated from ePTFE or Dacron, has been widely studied. Over the years, heparinized grafts have been tested clinically for infrainguinal (Walluscheck et al., 2005), femoropopliteal (Devine et al., 2001, Devine et al., 2004; Bosiers et al., 2006; Daenens et al., 2009; Lindholt et al., 2011), and aortic bypass grafting (Tsilimparis et al., 2015), and as arteriovenous shunts (Shemesh et al., 2015). Overall, trials have been successful, with patency rates comparable to native saphenous vein, and improved over non-heparinized synthetic grafts. An example illustrating this improvement is provided in Figure 4, which shows the luminal surface of a GORE graft with and without their CARMEDA BioActive Surface (CBAS^®^ heparin surface) after 2 h of implantation in a canine carotid artery model (Biran and Pond, 2017). The graft without the heparin coating has a clear thrombotic occlusion, compared to the graft with the CBAS heparin surface, which does not have any apparent thromboses. In addition to its established effects, it may be possible that heparin traps circulating growth factors, which may further enhance endothelialization and improve patency rates.

Due to the success of these studies, some heparinized grafts, such as the GORE PROPATEN graft, are now commercially available for bypassing large diameter vessels and some smaller diameter lower limb vessels. There have been mixed reports concerning long term (5 years) patency rates compared to grafts without heparin, which may be due to different immobilization methods (Devine et al., 2004; Samson et al., 2016). Covalent immobilization techniques maintain heparin in its bioactive state tethered to the graft, with limited release over time (Larm et al., 1983). Other immobilization or coating methods may result in faster heparin release, limiting its long-term protective effects.

### Growth Factor Delivery

Growth factors have been tested to enhance endothelialization of synthetic vascular grafts since the 1990s (Greisler et al., 1992; Gosselin et al., 1996; Doi and Matsuda, 1997). They can either be incorporated within coatings on the luminal surface of the graft, or immobilized directly to the graft surface. Coatings are more commonly used, as direct immobilization of growth factors onto synthetic surfaces can be challenging, and additional surface modifications may be required (Crombez et al., 2005). Fibrin and fibronectin are the most widely used coatings to incorporate growth factors, as these proteins alone directly bind to ECs, aiding in their adhesion (Zilla et al., 1989; Meinhart et al., 2005; Dahl et al., 2011; De Visscher et al., 2012).

Early studies incorporated FGF into fibrin glue coatings for ePTFE grafts. This appeared to enhance endothelialization in rabbits, though the potent effects of FGF on SMC proliferation also increased neointimal growth (Greisler et al., 1992). Later, growth factors more specific to ECs, such as VEGF, were tested. VEGF is well-established to attract ECs and stimulate EC proliferation (Ferrara and Devis-Smyth, 1997). When incorporated into a Matrigel coating, VEGF resulted in enhanced endothelialization, but also increased intimal hyperplasia in a rat model (Randone et al., 2005). Similar increases in neointimal growth were observed when VEGF was incorporated into fibrin-coated grafts implanted in pigs (Walpoth et al., 2007). This may be because VEGF can stimulate ECs to produce other growth factors, including FGF, that increase SMC proliferation (Randone et al., 2005). Thus, VEGF alone may not be an ideal growth factor for this application.

Stromal-cell derived factor-1α has been used to stimulate stem cell recruitment and accelerate healing. When implanted in sheep, grafts with SDF-1α incorporated into a fibronectin coating had less intimal growth compared to controls (De Visscher et al., 2012). Other growth factors may be effective for enhancing endothelialization and preventing intimal hyperplasia, but have been largely studied on decellularized native arteries rather than synthetic grafts, and are discussed in more detail in Section “Decellularized Grafts for Growth Factor Delivery” below. Overall, growth factor coatings have been less successful than heparin immobilization. It is likely that combinations of growth factors and other molecules will be needed to both enhance endothelialization and prevent stenosis.

### Other Molecules for Enhanced Endothelialization

Antibodies specific to ECs may also be effective for enhancing EC adhesion to synthetic surfaces. CD133 and VEGF receptor-2 are both specific to endothelial progenitor cells (EPCs). Lu et al. (2013) incorporated CD133 antibodies into a collagen coating layered with heparin, and observed enhanced endothelialization in a porcine model (Lu et al., 2013). Grafts coated with VEGF receptor-2 antibodies have also shown promise, leading to increased adhesion of VEGF receptor-2-expressing cells in *in vitro* studies (Liebler et al., 2016). Sapienza et al. (2009) coated the luminal surface of grafts with antibodies against endogenous FGF, PDGF, and TGF-β1. The growth factors bind to these antibodies, which prevents them from activating local growth factor receptors and stimulating SMC proliferation. This resulted in less intimal hyperplasia in a 4 weeks pig study (Sapienza et al., 2009), though longer term studies are needed to ensure that the growth factor inhibition does not harm the endothelium. Additional approaches for enhancing EC adhesion on synthetic grafts are reviewed in (Melchiorri et al., 2013; Ren et al., 2015).

In addition to immobilization or incorporation within coatings, some molecules can be incorporated within synthetic grafts during fabrication. Ishii et al. (2008) incorporated sirolimus and heparin into polycarbonate-siloxane polyurethane during the fabrication process. These grafts showed increased endothelialization and reduced intimal growth compared to grafts with either drug alone in a rabbit model (Ishii et al., 2008).

The controlled release of heparin and other therapeutics has made synthetic vascular grafts a viable alternative for several bypass grafting applications. However, there have still not been any reports of synthetic grafts with acceptable patency rates for small diameter high-flow arteries such as the coronary artery. Further optimization of therapeutic agents used and their release kinetics may help address this need. However, mechanical properties also play a critical role in graft success, as compliance mismatch is well-established to trigger restenosis (Abbott et al., 1987). Utilizing growth factor release to stimulate endothelialization and tissue ingrowth, in addition to optimizing graft compliance may lead to higher patency rates.

## Controlled Release From Tissue Engineered Blood Vessels

Tissue engineered blood vessels can be fabricated in a variety of ways. A common approach is to seed cells onto natural or synthetic polymer scaffolds and allow the construct to mature and remodel in a bioreactor (Niklason et al., 1999; Grassl et al., 2002). Alternatively, TEBVs can be fabricated via cellular self-assembly approaches, where constructs are fabricated entirely from cells and their secreted extracellular matrix (L’Heureux et al., 1998; Gwyther et al., 2011). While these approaches have had some success, many challenges remain, such as establishing a healthy, contractile SMC phenotype, optimizing graft strength and compliance, and achieving complete endothelialization. Localized and controlled bioactive factor delivery may be able to address some of these problems.

The goal of many tissue engineering strategies is for native tissue to ultimately replace the original synthetic or natural polymer construct. To do this, the scaffold degradation rate must be carefully engineered to match the rate of new tissue growth. Thus, depending on the system, growth factors released in TEBVs may be designed to mature the graft and enhance cell proliferation, matrix deposition, or cell differentiation, compared to drug-eluting devices discussed earlier, which are predominantly designed to prevent SMC proliferation and new tissue growth. Therefore, it is important to consider how different types of TEBVs will be integrated with host tissue when designing controlled release systems.

### Decellularized Grafts for Growth Factor Delivery

Decellularized allogeneic vessels have had some clinical success and are commercially available as alternatives to synthetic grafts for small diameter vessels when autologous grafts are not available (O’Banion et al., 2016). However, these grafts still have low patency rates (56% primary patency at 1 year for infrapopliteal bypass), primarily due to thrombosis (Randon et al., 2010). As with synthetic vascular grafts, molecules such as heparin may be used to enhance endothelialization and reduce the risk of thrombosis and restenosis (Conklin et al., 2002; Wang et al., 2007; Zhou et al., 2012; Dimitrievska et al., 2015). Growth factors immobilized on graft surfaces have also been studied (Conklin et al., 2004; Zeng et al., 2010; Zeng et al., 2012). Specifically, grafts with immobilized brain-derived neurotrophic factors (Zeng et al., 2012) and nerve growth factor (Zeng et al., 2010) increased endothelial progenitor cell recruitment and improved endothelialization and patency in rat models. While growth factor coated decellularized allogeneic blood vessels are a promising alternative as small-diameter bypass grafts, their limited availability may ultimately prevent widespread use.

### Degradable Electrospun Polymer Scaffolds

Degradable polymer scaffolds can enable highly tuned release of growth factors and other therapeutics. Polymers can be designed and fabricated with customizable degradation rates, which play a major role in release kinetics. Growth factor incorporation in degradable polymeric TEBVs has been mainly limited to electrospinning approaches. Other types of degradable TEBV scaffolds may also be ideal for this application, but have been largely unexplored. These are restricted to a small number of studies where growth factors were tethered to degradable polymer surfaces, rather than directly incorporated within the scaffolds (Edlund et al., 2011; Melchiorri et al., 2015). In this section, we will focus on molecules that are directly incorporated within electrospun TEBVs during fabrication, rather than tethered onto their surface.

Electrospinning is a process where an electric current is applied to a polymer solution, which forms thin micro- or nano-fibers that accumulate to create scaffold. Electrospun scaffolds are advantageous because they have a porous structure that provides a high surface area to volume ratio. This allows for potential sustained delivery, as encased molecules must first diffuse out of the polymer fiber, and then through the pores in the graft. With electrospinning, parameters that may affect bioactive molecule release, such as polymer composition, fiber diameter, and pore size, can be easily tuned by adjusting manufacturing parameters.

**FIGURE 5:**
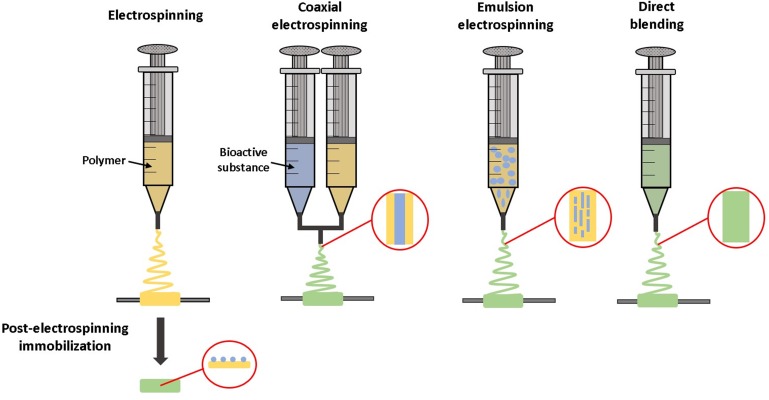
Techniques for incorporating bioactive molecules within electrospun grafts. Molecules can be tethered to the material after electrospinning. With coaxial electrospinning, the material and bioactive substance are combined during the electrospinning process, while being dispensed from two separate syringes. Alternatively, with emulsion electrospinning or direct blending a solution of bioactive molecules is mixed with the material prior to electrospinning.

There are several methods for incorporating molecules within electrospun fibers, reviewed in Rychter et al. (2017) and summarized schematically in Figure 5. Direct blending is used when the polymer and growth factor have similar solubility. The polymer and therapeutic are combined prior to electrospinning to form one solution, which is advantageous due to simplicity in manufacturing. However, this may result in a high initial burst release, as drugs and growth factors tend to be located predominantly on the fiber surface (Kim et al., 2004). With emulsion electrospinning, a typically aqueous solution of growth factor is emulsified in a solution of polymer, which leads to droplets of growth factor solution becoming encapsulated within a polymer shell after electrospinning. Coaxial electrospinning utilizes two different pumps to enable electrospinning of two different solutions together to create core-shell fibers, where a growth factor solution core is contained in a polymer shell (Rychter et al., 2017).

Electrospinning has been used to incorporate a wide range of molecules into vascular grafts (summarized in Table 4). Many of these designs have advanced to pre-clinical testing, including grafts eluting VEGF to enhance endothelialization (Zhang et al., 2013), heparin to prevent thrombosis (Huang et al., 2013; Yao et al., 2014; Spadaccio et al., 2016), platelet-derived growth factor (PDGF) to encourage SMC ingrowth (Zhang et al., 2013), SDF-1α to recruit progenitor cells (Yu et al., 2012), paclitaxel to inhibit restenosis (Innocente et al., 2009), and antibiotics to prevent infection (Liu et al., 2015). Other molecules, such as steroids (Chen et al., 2015) and anti-thrombotic drugs (Punnakitikashem et al., 2014) have shown promise in *in vitro* studies for decreasing inflammation and thrombosis, but have not progressed to *in vivo* testing.

**Table 4:** Molecules delivered in synthetic and tissue engineered vascular grafts.

Molecule	Delivery mechanism	Effects
Heparin	Synthetic non-degradable grafts, degradable TEBVs, decellularized allografts	Thrombosis prevention
FGF	Synthetic non-degradable grafts	Increase endothelialization
VEGF	Synthetic non-degradable grafts, TEBVs	Increase endothelialization
SDF-1α	Synthetic non-degradable grafts, TEBVs	Accelerate healing, recruit progenitor cells
PDGF antibody	Synthetic non-degradable grafts	Prevent SMC proliferation
TGF-β1 antibody	Synthetic non-degradable grafts	Prevent SMC proliferation
FGF antibody	Synthetic non-degradable grafts	Prevent SMC proliferation
CD133 antibody	Synthetic non-degradable grafts	Increase endothelialization
VEGF-receptor 2 antibody	Synthetic non-degradable grafts	Increase endothelialization
Brain-derived neurotrophic factors	Decellularized allografts	Increase endothelialization
Nerve growth factor	Decellularized allografts	Increase endothelialization
PDGF	TEBVs	Increase SMC ingrowth
Paclitaxel	TEBVs	Inhibit SMC proliferation and restenosis
Osteopontin-derived peptide	TEBVs	Increase EC adhesions
TGF-β1	TEBVs	Increase SMC differentiation

*Summary of molecules discussed in Sections “Targeted Delivery in Vascular Grafts” and “Controlled Release From Tissue Engineered Blood Vessels,” their delivery mechanisms, and effects*.

Grafts with incorporated heparin have shown promise in canine studies. Wang et al. (2013) incorporated heparin into poly(L-lactide-co-epsilon-caprolactone) electrospun grafts by coaxial electrospinning. With the addition of a pre-endothelialization step, they achieved an 89% patency rate when heparin-eluting grafts were implanted in a canine femoral artery for 24 weeks (Wang et al., 2013). However, grafts that were not pre-endothelialized had very poor patency, even with incorporated heparin (Wu et al., 2015).

As with other drug delivery applications, single therapeutic approaches are often unsuccessful. Dual delivery approaches may be more effective for addressing the multiple challenges associated with implanting TEBVs. Zhang et al. (2013) developed a two-layer electrospun vascular graft, with an inner VEGF-loaded layer to induce endothelialization, and an outer PDGF-loaded layer to stimulate SMC infiltration and new tissue growth. Grafts remained patent for 4 weeks after implantation in rabbit carotid arteries (Zhang et al., 2013). A similar system described in Campagnolo et al. (2016) incorporated an osteopontin-derived peptide in the luminal side to increase EC adhesion, and a heparin-binding peptide on the abluminal side to trap secreted VEGF and further enhance EC migration and proliferation. *In vitro* tests showed promising increases in EC adhesion, although this system has not yet been tested *in vivo* (Campagnolo et al., 2016).

In the future, customizing the delivery of therapeutics for individual patients may also help improve graft success, as different patients may experience different rates of healing and endothelialization. Wang et al. (2015) developed an enzyme-functionalized vascular graft that locally produced nitric oxide (NO) when a prodrug was administered systemically in rats. After 30 days, explanted NO-producing grafts had enhanced function, endothelialization, and reduced platelet adhesion compared to grafts that did not produce NO (Wang et al., 2015). This system may allow for the further tuning of molecule delivery, as the molecule can locally be produced “on demand” only when the patient needs it.

Overall, electrospinning is a well-suited graft fabrication technique to incorporate a variety of different growth factors and therapeutics into polymer scaffolds with highly tunable release kinetics. However, while pre-clinical trials have been promising, most still exhibit high stenosis rates that have prevented grafts from moving forward to clinical trials. Optimizing delivery of therapeutics may improve success. As with permanent synthetic grafts, therapeutic delivery in combination with altering other graft properties, such as compliance, may be critical for improving overall patency. Additionally, parameters such as pore size and fiber diameter may need to be optimized to achieve adequate cellular infiltration and tissue ingrowth. Electrospun grafts are reviewed in greater detail in Rychter et al. (2017).

### Microsphere-Mediated Growth Factor Delivery in Engineered Vascular Tissue

Microsphere (MS)-mediated growth factor delivery has been used for years to mature and differentiate many engineered tissues, including cartilage (Solorio et al., 2010), bone (Wang et al., 2009), and stem cell aggregates (Carpenedo et al., 2009; Solorio et al., 2010; Bratt-Leal et al., 2013). MS incorporation alone can increase tissue strength (Solorio et al., 2012b; Dikina et al., 2015), oxygen diffusion (Hayashi and Tabata, 2011; Tajima and Tabata, 2013), cell viability (Garcia Cruz et al., 2013; Tajima and Tabata, 2013), and uniformity of matrix deposition (Solorio et al., 2012a). However, their application in vascular tissue engineering has been limited. Others have incorporated gelatin MS into cell spheroids, which were fused into vascular tissue, but the MS primarily served to stabilize the construct and were not used for growth factor delivery (Twal et al., 2014). Our group has demonstrated that MS loaded with TGF-β1 can be used to increase SMC contractile protein expression within self-assembled SMC rings (Strobel et al., 2017). This approach may be well-suited for applications where systemic or exogenous delivery of a growth factors may be harmful or not possible, or for thick, high cell density engineered tissues where growth factors cannot diffuse through the entire construct.

### Future of Growth Factor Delivery in TEBVs

There is great potential for growth factors to be incorporated into other systems, such as TEBVs fabricated from hydrogels or other polymer scaffolds, which have yet to be explored. Incorporation within scaffolds may help overcome diffusion limitations and result in more uniform treatment than exogenous growth factor delivery. It may also allow for continuous delivery of growth factors after TEBV implantation, when exogenous or systemic delivery is not feasible. Many different types of molecules may be utilized, depending on the application. As discussed above, cytokines for EC recruitment have proven essential for preventing thrombosis and intimal hyperplasia. Other growth factors such as PDGF and FGF may be able to stimulate new tissue formation in degrading polymer grafts, and TGF-β1 may be used to differentiate SMCs and drive TEBV maturation.

Tissue maturation may further be enhanced by adjustments to growth factor release kinetics. Release of multiple growth factors sequentially may be beneficial for vascular tissue engineering. Gong and Niklason (2008) developed an optimized procedure for fabricating TEBVs by treating them *in vitro* with exogenous PDGF for 4 weeks to stimulate new tissue growth, and then TGF-β1 for 4 weeks to promote differentiation. This sequential delivery could also be obtained by designing biomaterials for controlled dual-delivery, an approach that is already being used to create microvasculatures within other engineered tissues (Chen et al., 2007; Freeman and Cohen, 2009).

Delayed release of anti-inflammatory molecules days or weeks after TEBV implantation is another potential application of controlled release. While the inflammatory response may be critical immediately following implantation, delayed release of anti-inflammatory molecules may prevent problems such as intimal hyperplasia from occurring later (Washington and Bashur, 2017).

Spatiotemporal control of the release of multiple growth factors may also be applied to modeling focal vascular diseases. Because many vascular diseases such as atherosclerosis, intimal hyperplasia, and aneurysm affect only one region of the vessel, delivering growth factors or other molecules specifically within a focal region within TEBVs may enable the creation of such models. This could potentially be accomplished with modular TEBV approaches and controlled release systems (Strobel et al., 2018). Spatially controlled release may also be advantageous for culturing and maintaining distinct tissue phenotypes in multi-tissue constructs such as trachea, which have alternating smooth muscle and cartilage regions (Dikina et al., 2018).

## Regulatory Challenges

There are many challenges when designing an effective drug-eluting medical device or tissue engineered construct. In addition to design challenges, regulatory approval of these products is a major consideration. Medical devices, drugs, and biologics are all regulated by the Food and Drug Administration in the United States, to ensure safety and efficacy of any new product. Drug-eluting stents pose a regulatory challenge because they contain both a device and a drug, making them a combination product. However, they have now been in use for many years, and regulatory guidelines are well-established [described in detail in Food and Drug Administration [FDA] (2008)]. Because there are many approved drug-eluting stent designs, and they often utilize already approved drugs, new designs may have a more clear pathway to approval, although extensive *in vitro*, pre-clinical, and clinical studies are still required. However, experimental designs and therapeutics, such as siRNA eluting stents, may be considered new drugs and require even more thorough safety and efficacy testing.

The wide range of nanoparticle designs can make them more challenging to regulate. For example, size may play a critical role in the safety of some nanoparticle-based therapeutics, but have no effect on the safety of others, depending on the material and delivery and targeting mechanisms. Nanoparticles are usually regulated under the same standards as other drugs. However, because there may be additional concerns about toxicity, clearance, and biodistribution, some additional recommendations are in place [detailed in Food and Drug Administration [FDA] (2017)]. The largest barrier to approval is the often limited understanding of nanoparticle pharmacokinetics. Improvements in pharmacokinetic modeling may help accelerate the approval of these therapies in the future. Regulation of nanoparticles is reviewed in detail in Narang et al. (2013).

Tissue engineered blood vessels provide a unique regulatory challenge, because they are a relatively new technology that combines the regulatory umbrellas of biologics, tissues, and devices. While ASTM and ISO standards exist for testing synthetic vascular grafts (American National Standard [ANS], 2010) and some other tissue engineered products, none specifically cover the characterization and manufacturing of TEBVs (Lee et al., 2010). With TEBVs, additional factors need to be considered such as how cells will affect scaffold properties over time. Because cells, materials, scaffolds, and eluted molecules are produced separately, quality control testing may need to be performed both before and after TEBV fabrication. Deciding at which timepoints during TEBV maturation and remodeling that they should be tested can also be a challenge (Lee et al., 2010). As more TEBVs move forward into clinical trials, more regulatory guidelines will be established to help address these challenges.

## Summary and Unmet Challenges

Localized bioactive molecule delivery has enormous potential for more effective and safe treatment of cardiovascular diseases than systemic treatments. Drug-eluting stents are already widely used clinically and have shown great success. Other systems, such as bioactive molecule-eluting nanoparticles and aneurysm coils, still have some challenges to overcome before they can be used clinically. While these fields have made tremendous progress, there is only a limited ability to predict how effective new designs will be *in vivo*. While computational modeling has improved our ability to predict release curves, these models are still highly limited. Patient-to-patient variability in the inflammatory and immune responses, thrombus formation, healing, and re-endothelialization makes this an exceptionally challenging problem, as these factors can affect material degradation, molecule release, and diffusion into the tissue. In the future, more advanced models may further improve our ability to streamline drug-eluting medical device design and maximize the patient benefit of these products. Existing drug-release models are often based on animal studies, which may not translate to humans. It is possible that TEBVs may in the future be used to better mimic the human *in vivo* environment *in vitro*, to create a more realistic system for modeling drug release profiles. TEBVs may also allow for the fabrication of patient-specific disease models, which may further improve our ability to account for patient-to-patient variability.

Combining nanoparticle and drug-eluting stent approaches may in the future allow for sequential delivery of multiple factors, allowing for more comprehensive, customized treatments. The enormous number of nanoparticle systems and drug-eluting stents in development may ultimately provide physicians with a wide range of options, enabling them to customize targeted drug delivery for each individual patient’s needs. Future work in the field may focus on combining several technologies to deliver multiple therapeutics sequentially, and to allow for such customization.

Vascular bypass grafts have made significant progress as alternatives to autologous grafting for large and medium blood vessel bypass procedures, but progress has been slow for small diameter applications. TEBVs for coronary artery bypass grafting have achieved success in pre-clinical studies, but have yet to be applied clinically. Some of the challenges currently facing the field include optimization and *in vivo* measurement of drug release kinetics, the need for multi-drug approaches with differing release profiles, problematic compliance mismatch between vascular grafts and native vessels, and insufficient tissue ingrowth into vascular grafts and TEBVs. As with other vascular interventions, future work in tissue engineering may focus on optimizing biomaterials to release multiple growth factors or other molecules, to fabricate and mature tissue into biomimetic grafts.

## Author Contributions

HS is the primary author. EQ created the figures and assisted with literature searches. EA revised the structure and content, and edited the final draft. MR advised HS and EQ on structure and content, and edited the manuscript.

## Conflict of Interest Statement

The authors declare that the research was conducted in the absence of any commercial or financial relationships that could be construed as a potential conflict of interest.
